# Impact of posttransplant cyclophosphamide on the outcome of patients undergoing unrelated single-unit umbilical cord blood transplantation for pediatric acute leukemia

**DOI:** 10.1186/s12885-022-10309-9

**Published:** 2022-11-18

**Authors:** Xin-Yu Li, Li-Ping Zhan, Dian-Dian Liu, Xia-Wei Han, Han Chen, Zheng-Zhou Wu, Yin Wang, Li-Ping Que, Xiao-Jun Wu, Su Liu, Kai-Mei Wang, Shao-Liang Huang, Jian-Pei Fang, Ke Huang, Hong-Gui Xu

**Affiliations:** 1 Department of Pediatrics, Sun Yat-Sen Memorial Hospital, Sun Yat-Sen University, Guangzhou, 510120 China; 2 Guangdong Provincial Key Laboratory of Malignant Tumor Epigenetics and Gene Regulation, Sun Yat-Sen Memorial Hospital, Sun Yat-Sen University, Guangzhou, 510120 China; 3 Children’s Medical Center, Sun Yat-Sen Memorial Hospital, Sun Yat-Sen University.No, 107, West Yan-Jiang Road, Guangzhou, 510120 Guangdong China

**Keywords:** Umbilical cord blood transplantation, Acute leukemia, Posttransplant cyclophosphamide

## Abstract

**Background:**

Umbilical cord blood transplantation (UCBT) from unrelated donors is one of the successful treatments for acute leukemia in childhood. The most frequent side effect of UCBT is peri-engraftment syndrome (PES), which is directly associated with the greater prevalence of acute and chronic graft-versus-host-disease (aGvHD and cGvHD). In haploidentical stem cell transplantation, posttransplant cyclophosphamide (PTCY) has been demonstrated to be an effective method against GvHD. However, the effects of PTCY as a GvHD prophylactic in UCBT had not been investigated. This study aimed to evaluate the effects of PTCY on the outcomes of UCBT for pediatric acute leukemia.

**Methods:**

This retrospective study included 52 children with acute leukemia who underwent unrelated single-unit UCBT after myeloablative conditioning regimens. The results from the PTCY and non-PTCY groups were compared.

**Results:**

The incidence of transplantation-related mortality in non-PTCY and PTCY were 5% and 10% (*p* = *0.525*), respectively. The incidence of relapse in non-PTCY and PTCY were 5% and 23% (*p* = *0.095*), respectively. Second complete remission status (CR2) was an independent risk factor for relapse-free survival (*hazard ratio* = 9.782, *p* = *0.001*). The odds ratio for sepsis or bacteremia incidence was significantly greater in the PTCY group (9.524, *p* = *0.017*). PTCY group had increased rates of cytomegalovirus activity and fungal infection. The incidence of PES, aGvHD, cGvHD, and hemorrhagic cystitis in the PTCY group was lower than that in the non-PTCY group, although it was not significantly different. Additionally, higher doses of PTCY (29 mg/kg and 40 mg/kg) were associated with lower incidences of aGvHD and severe GvHD (65% and 29%, respectively) than lower doses (93% and 57%, respectively). Engraftment time and graft failure incidence were similar across groups.

**Conclusion:**

The results support the safety and efficiency of PTCY as part of PES controlling and GvHD prophylaxis in single-unit UCBT for children with acute leukemia. A PTCY dosage of 29 mg/kg to 40 mg/kg appears to be more effective in GvHD prophylaxis for UCBT patients.

**Supplementary Information:**

The online version contains supplementary material available at 10.1186/s12885-022-10309-9.

## Introduction

Unrelated umbilical cord blood transplantation (UCBT) is one of the effective treatments for hematological malignant diseases, and it has an advantage for those who need urgent transplantation because of its immediate availability from cord blood (CB) banks. Peri-engraftment syndrome (PES) [1–4], especially severe PES, is associated with the higher incidence of acute graft-versus-host-disease (aGvHD) and chronic graft-versus-host-disease (cGvHD) [1, 3, 5–7]. Severe aGvHD is associated with high overall mortality and NRM in pediatric single UCBT [8]. However, mild PES reduces the relapse rate in acute myeloid leukemia (AML) after UCBT [9]. Therefore, to be able to moderate the severity of PES has been an important issue in the UCBT study.

The incidence of PES can be decreased by early immunosuppression after UCBT [10–12], as well as by tocilizumab, the interleukin-6 (IL-6) antibody [13], which needs further clinical trial and long-term observation for outcomes. In previous studies on UCBT [1, 14, 15], GvHD prophylaxis consisted of cyclosporine A (CsA) or tacrolimus (Tac) in combination with methotrexate (MTX) or corticosteroid has been used in children [16]. MTX has been reported to decrease the incidence and severity of PES. However, the optimal MTX dosage in UCBT remains not available [10]. Another treatment, anti-thymocyte globulin (ATG), has been used in UCBT for children with leukemia. However, ATG often results in delayed and poor T-cell reconstitution, which leads to high incidences of infection and related mortality [17]. Therefore, it is necessary to explore new immunosuppression strategies for PES control and GVHD prophylaxis in UCBT.

Recently, posttransplant cyclophosphamide (PTCY) is one of the most widely used regimens for GvHD prophylaxis in Haplo-HSCT [18, 19], and has the treatment not increase the incidence of graft failure (GF) and relapse. Compared with standard double doses of PTCY (50 mg/kg on day + 3 and day + 4), a single dose of PTCY (50 mg/kg on day + 3) had a similar effect in preventing aGvHD for Haplo-PBSCT patients [18]. Furthermore, PTCY presents lower incidences of viral and fungal infection than ATG-based regimens [20], making PTCY a potentially better candidate for PES control and GvHD prophylaxis in UCBT.

However, the impact of PTCY in UCBT for acute leukemia had not been studied. Therefore, we investigated PTCY as a GvHD prophylaxis in UCBT patients. By retrospectively analyzing the clinical data of UCBT in children with acute leukemia, this report evaluated the efficacy and safety of the PTCY in UCBT.

## Subjects and methods

### Patients and donors

Fifty-two patients who received UCBT in the Department of Pediatrics of Sun Yat-sen Memorial Hospital between August 2018 and February 2021 were included in this study. These patients are with AML, acute lymphoblastic leukemia (ALL), or mixed lineage leukemia (MLL). The median follow-up was 21.6 months (range, 1.8 to 38.2 months). The latest follow-up was on March 1, 2022. The characteristics of patients are summarized in Table 1. Between August 2018 and August 2019, 21 patients received non-PTCY prophylaxis against GvHD using either CsA or Tac in combination with Mycophenolate Mofetil (MMF) from day + 1 (non-PTCY group). Between August 2019 and February 2021, 31 patients received CsA or Tac with PTCY on day + 3 and day + 5 (PTCY group).

**Table 1:** Patients’ characteristics and the comparison for characteristics and outcomes in PTCY and non-PTCY groups

		Total (*n* = 52)	Group		*p value*
			PTCY (*n* = 31)	non-PTCY (*n* = 21)	
Age, years		5.8 (1.1,13.5)	6.04 (SD = 3.13)	6.15 (SD = 3.47)	*0.913*
Sex, n	male	37	23	14	*0.557*
	female	15	8	7	
Weight, kg		17.6 (9.0, 42.4)	19.35 (SD = 7.47)	18.60 (SD = 6.35)	*0.707*
Primary diagnosis, n	AML	27	19	8	*0.259*
	ALL	23	11	12	
	others	2	1	1	
Remission status, n	CR1	47	27	20	*0.329*
	CR2	5	4	1	
HLA matching, n	7/10	4	3	1	*0.704*
	8/10	22	14	8	
	9/10	17	10	7	
	10/10	9	4	5	
HLA matching 2, n	7/10 or 8/10	26	17	9	*0.329*
	9/10 or 10/10	26	14	12	
TNC, 10^7^/kg (range)		6.15 (3.3, 15.3)	6.78 (5.5, 8.06)	6.31 (3.29, 11.15)	*0.801*
CD34^+^, 10^5^/kg (range)		2.61 (0.72, 11.6)	3.25 (1.26, 10.7)	1.97 (0.72, 11.6)	*0.003*
Follow-up, months (range)		21.6 (1.8, 38.2)	14.4 (1.8, 30.0)	34.3 (2.2, 38.1)	< *0.001*
Neutrophil engraftment, days (range)		14 (11, 22)	15.8 (14.6, 16.9)	14.5 (11,19)	*0.160*
PLT engraftment, days (range)		32 (12, 61)	31.9 (27.8, 35.9)	34.5 (13, 52)	*0.233*
Graft failure, n (%)		2 (4)	1 (3)	1 (5)	*0.777*
PES, n (%)		45 (87)	25 (81)	20 (95)	*0.130*
Corticosteroid responding PES, n (%)		38 (73)	17 (71)	19 (95)	*0.054*
Acute GvHD, n (%)		42 (81)	24 (77)	18 (86)	*0.456*
Grade 3 and 4 Acute GvHD, n (%)		20 (38)	13 (42)	7 (33)	*0.532*
HC, n (%)		14 (27)	7 (23)	7 (33)	*0.431*
Chronic GvHD, n (%)		24 (46)	13 (42)	11 (52)	*0.458*
EBV activity, n (%)		1(2)	0 (0)	1 (5)	*0.404*
CMV activity, n (%)		6 (12)	5 (16)	1 (5)	*0.211*
pneumonia, n (%)		17 (33)	10 (32)	7 (33)	*0.584*
Fungal infection, n (%)		8 (15)	7 (23)	1 (5)	*0.081*
Sepsis or bacteremia, n (%)		11 (21)	10 (32)	1 (5)	*0.017*
TRM, n (%)		4 (8)	3 (10)	1 (5)	*0.514*
Relapse, n (%)		7 (13)	6 (19)	1 (5)	*0.130*
Event-free survival, n (%)		41 (79)	22 (71)	19 (91)	*0.091*

*PTCY* posttransplant cyclophosphamide, *SD* standard deviation, *AML* acute myeloid leukemia, *ALL* acute lymphoblastic leukemia; others, mixed lineage leukemia, *CR1* first complete remission, *CR2* second complete remission, *HLA* human lymphocyte antigen, *TNC* total nucleated cell, *CD34*^+^ CD 34^+^ cell counts, *PLT* Platelet, *GvHD* Graft-versus-host-disease, *HC* Hemorrhagic cystitis, *EBV* Epstein-Barr virus, *CMV* Cytomegalovirus, *PES* peri-engraftment syndrome, *TRM* Transplantation related mortality

Donors were unrelated CB from public cord blood banks in mainland China. When searching for unrelated CB, complete human leukocyte antigen (HLA) matches between 10 loci of the ‐A, ‐B, ‐C, ‐DR, and ‐DQ alleles were required, with at least 7 out of 10 loci matching at high-resolution level. Two mismatch loci were not permitted to be located at the same allele.

### Conditioning regimens and GvHD prophylaxis

**Fig. 1:**
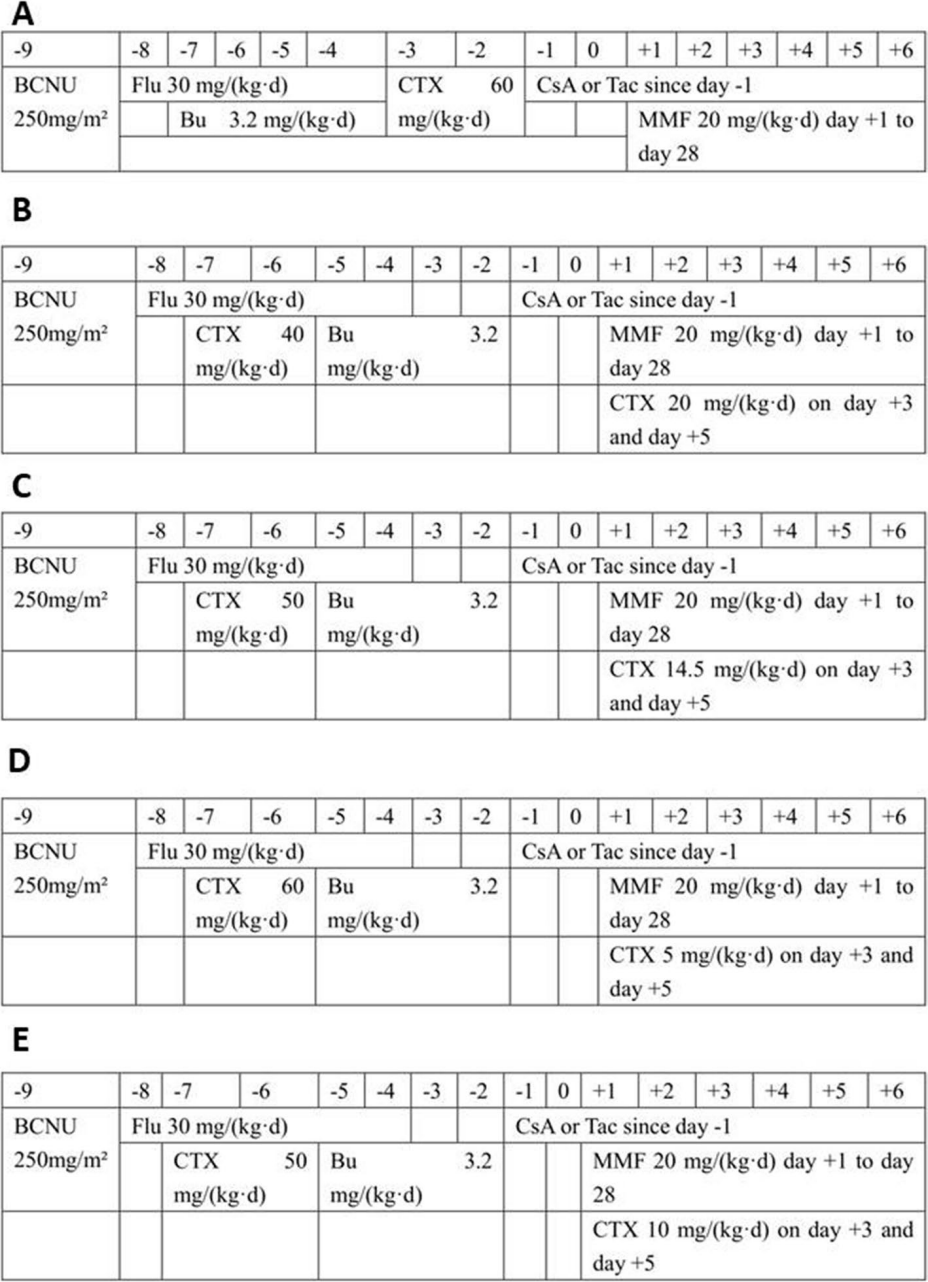
Conditioning regimens. **A** Conditioning regimen of non-PTCY group. **B**-**E** Conditioning regimens of PTCY group

The conditioning regimen of the non-PTCY group consisted of cyclophosphamide (CY) at 60 mg/kg for 2 days, BU at 3.2 mg/kg for 4 days, and fludarabine (Flu) at 30 mg/m^2^ for 5 days (Fig. 1-A), with or without 250 mg/m^2^ semustine for one day. The conditioning regimen of the PTCY group consisted of CY at 40–60 mg/kg for 2 days before transplantation, BU at 3.2 mg/kg for 4 days, and Flu at 30 mg/m^2^ for 5 days (Fig. 1-B to E), with (*n* = 19) or without (*n* = 33) semustine at 250 mg/m^2^ for one day. Semustine was included in patients who were high-risk ALL and who had a history of central nervous system leukemia (CNSL).

All patients received GvHD prophylaxis of CsA at 3 mg/kg/day (*n* = 37) or Tac at 0.02 mg/kg/day (*n* = 15) as continuous infusion from day -1 in combination with MMF at 20 mg/kg/day from day + 1 to day + 28. CsA blood level was maintained at 150–250 ng/mL, and Tac blood level was maintained at 8–12 ng/mL. The 31 patients in the PTCY group received CY at 5–20 mg/kg/day on day + 3 and day + 5 (Fig. 1-B to E), among which, 7 patients received 20 mg/kg/day, 10 received 14.5 mg/kg/day, 3 received 10 mg/kg/day and 11 received 5 mg/kg/day on day + 3 and day + 5.

### Supportive care

Antibacterial prophylaxis was performed with intravenous piperacillin-sulbactam and antifungal prophylaxis was performed with intravenous micafungin, caspofungin, or oral posaconazole. Antiviral prophylaxis was performed with intravenous acyclovir from day -9 to day + 14, intravenous ganciclovir from day + 14 to day + 28, and oral valaciclovir from day + 28 to day + 120. Intravenous Immunoglobulin (IVIg) was given at 200–300 mg/kg every 14 days. Granulocyte colony-stimulating factor (G-CSF) was given intravenously at 5–10 mg/kg since day + 6. Oral mucositis was treated with local human recombinant interleukin-11 and parental nutrition. PES and aGvHD was treated with methylprednisolone. Cytomegalovirus (CMV) infection was treated with ganciclovir.

### Definitions

PES was diagnosed at the duration between the onset of clinical symptoms and neutrophil recovery according to the Spitzer Criteria (Supplementary Information 1). The grading of PES was done in accordance with previous literature [13].

CMV infection was diagnosed positive when blood CMV DNA copies exceeded 10^3^ copies/ml. Epstein-Barr virus (EBV) was diagnosed positive when blood EBV DNA copies exceeded 10^3^ copies/ml.

The modified Glucksberg grading of aGvHD [21] was applied in the diagnosis. The onset of aGvHD from preceding PES was defined as the day of neutrophil engraftment. Skin biopsies were not performed on any of the patients for diagnosis of PES or skin GvHD. The 2014 National Institutes of Health (NIH) consensus criteria [22] were used to diagnose and grade cGvHD.

No donor hematopoiesis beyond day + 28 was regarded as graft failure. Neutrophil engraftment and platelet engraftment were in accordance with the literature [18, 19]. Quantitative chimerism monitoring was performed by short-tandem repeat (STR)-based PCR techniques [23].

The safety end points of this study included transplantation-related mortality (TRM), overall survival (OS), event-free survival (EFS), relapse-free survival (RFS), and the incidence of relapse after UCBT. The efficiency end-points included the incidence of PES, aGvHD, cGvHD, and infections after UCBT.

Events included death, disease relapse, graft failure, cGvHD, and secondary malignancy. EFS was defined as the duration between UCBT and observation of events/the last contact. OS was defined as the duration between transplantation and death/the last contact. RFS was defined as the duration between UCBT and relapse, death, or last contact. The definition of TRM was death after UCBT except for death from disease relapse. Morphologic evidence of disease was defined as relapse.

### Immune cell recovery

Cells were analyzed as previously described in previous literature [24]. Fluorescence-conjugated monoclonal antibodies (BD multitest 6-color TBNK, San Jose, CA, USA) were added to mononuclear cells. Samples were analyzed on a Beckman navios cytometer (Beckman Coulter Life Science) and then analyzed using Navios tetra Software (Beckman Coulter Life Science). The lymphocyte subpopulation was gated and used as a reference for the determination of natural killer (NK) cell, total T-cell, CD3^+^CD4^+^ helper T-cell, CD3^+^CD8^+^cytolytic T-cell, and B-cell subsets between day + 28 and day + 35.

### Statistical analysis

All patient follow-up was done by outpatient service and telephone. Follow-up was updated on February 28, 2022. Patients without outpatient records within one month before the end of the study were confirmed by telephone follow-up. Loss of contact for over one month after transplantation was defined as lost contact. RFS and OS were calculated from the date of infusion of CB to the date of the first event. If no event was reported, the observation time would be recorded at the last follow-up. RFS and OS curves were estimated according to Kaplan–Meier with Greenwood’s standard error (SE) and they were compared by the two-tailed log-rank test. The Cox proportional-hazards regression model was used for multivariate analysis and Hazard Ratio (HR). Risk factors with a *p*-value < 0.1 in each univariate analysis were included in the multivariate analysis. The Mann–Whitney U-test was used for continuous variables from unpaired samples. The Chi-square test and Fisher’s exact probability test were used for the correlation and Odds Ratio (OR) analysis between two groups of data. Statistical analysis was carried out by using SPSS 23.0 (SPSS Institute, Cary, NC) and R. *p*-values < 0.05 were considered significant.

## Results

### Survival-related variants analysis

In this study, the OS and the RFS were 79% and 78%, respectively. Univariate analyses showed that HLA matching, with or without the use of PTCY, and remission status before transplantation were potential key factors for both OS and RFS, as displayed in Table 2. Further multivariate analysis found that remission status before transplantation was an independent risk factor for RFS, as displayed in Table 3.

**Table 2:** Univariate analysis of OS and RFS

Variables	OS, %		*p*	RFS, %		*p*
Diseases, mean(95%CI)						
AML	84.8 (71.1, 98.5)		*0.771*	85.2 (71.9, 98.5)		*0.358*
ALL	72.6(51.4, 93.8)			67.4(47.2, 87.6)		
HLA matching, mean(95%CI)						
10/10 and 9/10	95.7(87.3, 100)		*0.009*	96.2(88.8, 100)		*0.002*
8/10 and 7/10	70.7(52.1, 89.3)			59.3(39.5, 79.1)		
PTCY, mean(95%CI)						
Yes	70.9(51.5, 90.3)		*0.057*	67.5(49.3, 85.7)		*0.075*
No	89.9(76.6, 100)			90.5(77.5, 100)		
Remission, mean(95%CI)						
CR1	86.0(75.4, 96.6)		*0.011*	86.6(96.8, 76.4)		< *0.001*
CR2	60.0(17.1, 100)				0	

*AML* acute myeloid leukemia, *ALL* acute lymphoblastic leukemia; others, mixed lineage leukemia, *CR1* first complete remission, *CR2* second complete remission, *HLA* human lymphocyte antigen, *PTCY* posttransplant cyclophosphamide, *GvHD* graft-versus-host-disease, *PES* peri-engraftment syndrome, *OS* overall survival, *RFS* Relapse-free survival, *SE* Standard error, *95%CI* 95% confidence interval

**Table 3:** Multivariate analysis of OS and RFS

Variables	HR for OS (95%CI)	*p*	HR for RFS (95%CI)	*p*
HLA matching				
10/10 and 9/10	1	*0.071*	1	*0.417*
8/10 and 7/10	7.286(0.842, 63.044)		2.706(0.244, 29.967)	
PTCY				
No	1	*0.153*	1	*0.192*
Yes	4.342(0.581, 32.473)		4.178(0.488, 35.801)	
Remission				
CR1	1	*0.360*	1	*0.002*
CR2	1.986(0.457, 8.624)		21.042(3.158, 140.210)	

*HR* hazard ratio, *95% CI* 95% confidence interval, *CR1* first complete remission, *CR2* second complete remission, *HLA* human lymphocyte antigen, *PTCY* posttransplant cyclophosphamide, *GvHD* graft-versus-host-disease, *OS* overall survival, *RFS* relapse-free survival

### Comparison between the PTCY group and non-PTCY group

**Fig. 2:**
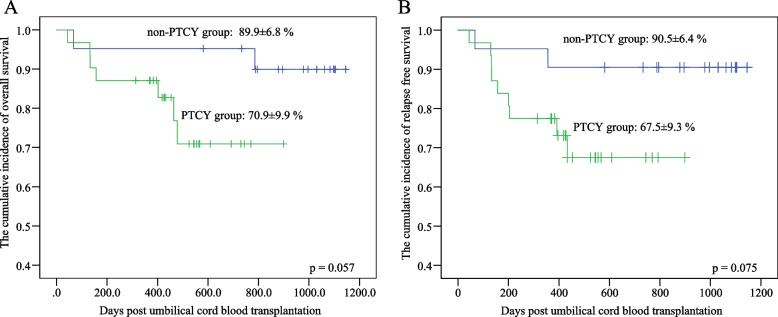
Comparison of Overall Survival and Relapse-Free Survival between PTCY group and non-PTCY group. **A** The cumulative incidence of overall survival of the PTCY and non-PTCY groups. **B** The cumulative incidence of relapse-free survival of PTCY and non-PTCY groups

To explore the impact of PTCY in UCBT, we compared the baseline and outcomes data in the PTCY and non-PTCY groups (Table 1). Even though the transfused CD34^+^ cell counts in both groups were significantly different (*p* = *0.003*), the cell count was not the risk factor affecting RFS (*p* = *0.674*). The HLA matching point counts of both groups were similar (*p* = *0.704*). Neutrophil engraftment time, platelet engraftment time, and graft failure incidence were similar across groups. Sepsis or bacteremia incidence in the PTCY group was significantly higher than in the non-PTCY group (OR = 9.524, 95% confidence interval (95%CI) (1.115–81.345), *p* = *0.017*). The rates of CMV activity and fungal infection were higher in the PTCY group (Table 1). The incidences of PES, aGvHD, cGvHD, and hemorrhagic cystitis (HC) in the PTCY group were lower than that in the non-PTCY group, although there was no significant difference (Table 1). The gradings of PES (*p* = *0.638*), HC (*p* = *0.407*), liver aGvHD (*p* = *0.316*), intestinal aGvHD (*p* = *0.178*), skin aGvHD (*p* = *0.410*), aGvHD (*p* = *0.871*) were similar between both groups. Survival curves for OS and RFS are provided in Fig. 2. The incidence of OS and RFS of both groups are displayed in Table 2. In the proportional hazards model for the sub-distribution of a competing risk, the TRMs of non-PTCY and PTCY were 5% (95% CI (4%, 5%)) and 10% (95% CI (9%, 10%)) (*p* = *0.525*), respectively, while the relapse rates were5% (95% CI (4%, 5%) and 23% (95% CI (21%, 24%)) (*p* = *0.095*), respectively. There were four deaths, one in non-PTCY group and three in PTCY group.

There were six cases of relapse in the PTCY group, among which, four patients were ALL. However, in the non-PTCY group, only one patient with ALL experienced relapse of CNSL. The median time of relapse of ALL was 204 days. The relapse rate of ALL was 22% (*n* = 5), while it was 7% in AML (*n* = 2) (*p* = *0.285*). The four cases of relapsed ALL used PTCY at the total dosage of 40 mg/kg. The incidence of relapsed ALL is significantly higher at a dosage of 40 mg/kg PTCY (4/7) than at other kinds of doses (*p* = *0.004*). In multivariate analysis, the risks of events in RFS for patients in the second complete remission (CR2) was 9.78 (95% CI (2.45, 39.12), *p* = *0.001*) after adjustment for HLA matching and PTCY usage. So, it remained to be determined whether the dose of PTCY affects the prognosis.

### Comparison between high-dose PTCY group and low-dose PTCY group

**Fig. 3:**
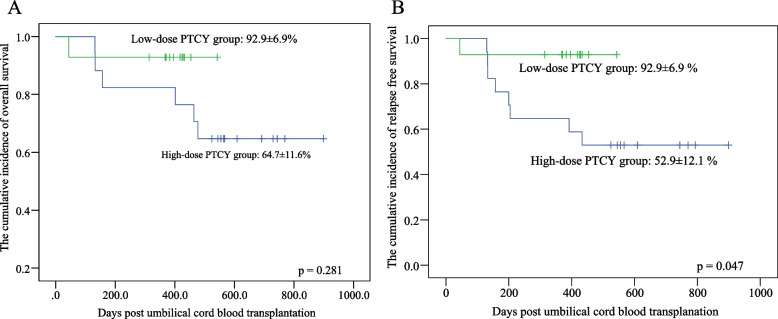
Comparison of Overall Survival and Relapse-Free Survival between high-dose PTCY group and low-dose PTCY group. **A** The cumulative incidence of overall survival of high-dose and low-dose groups. **B** The cumulative incidence of relapse-free survival of high-dose and low-dose groups

To further explore the impact of the dosage of PTCY in UCBT, we compared the baseline data and outcomes data between a high-dose PTCY group (40 mg/kg or 29 mg/kg) and a low-dose PTCY group (20 mg/kg or 10 mg/kg), as shown in Table 4. Interestingly, the incidences of aGvHD and severe GvHD in the high-dose PTCY group (65% and 29%, respectively) were both lower than the low-dose PTCY group (93% and 57%, respectively). However, the incidences of PES were not different across groups. No difference was found in complications, including CMV activation, EBV activation, fungal infection, and pneumonia. Incidence of sepsis or bacteremia was higher in the high-dose PTCY group, in which, 8/17 patients suffered from sepsis or bacteremia without leading to TRM. Increased relapse rate and decreased EFS were found in the high-dose PTCY group. There were six cases of relapse from the high-dose PTCY group. The relapse rate in the 40 mg/kg sub-group was 4/7 which contributed to 2/3 of the relapse cases. The other two cases of relapse were in the 29 mg/kg sub-group. No relapse was reported in the low-dose PTCY group. Survival curves for OS and RFS are provided in Fig. 3. The incidence of OS and RFS of both groups are displayed in Table 4. The incidence of TRM in the high-dose and low-dose groups were 13% and 7% (*p* = *0.515*), respectively. The incidence of relapse in high-dose PTCY and low-dose PTCY groups were 39% and 0 (*p* = *0.034*), respectively. In the multivariate analysis for RFS, the pre-transplantation remission status of CR2 was the independent risk factor (hazard ratio = 5.22, 95% CI (1.14, 23.87), *p* = *0.033*).

**Table 4:** Comparison for characteristics and outcomes of high-dose PTCY group and low-dose PTCY group

		high-dose PTCY group (*n* = 17)	low-dose PTCY group (*n* = 14)	*p value*
Age, years		6.12 (SD = 3.66)	5.96 (SD = 2.48)	*0.890*
sex, n	male	12	11	*0.613*
	female	5	3	
Weight, kg		19.7(SD = 9.0)	18.9 (SD = 5.3)	*0.759*
Primary diagnosis, n	AML	11	8	*0.436*
	ALL	6	5	
	others	0	1	
Remission status, n	CR1	14	13	*0.385*
	CR2	3	1	
HLA matching 2, n	7/10 or 8/10	11	6	*0.224*
	9/10 or 10/10	6	8	
TNC,10^7^/kg (range)		7.1 (3.39, 13.9)	6.8 (1.0, 15.3)	*0.795*
CD34^+^, 10^5^/kg (range)		3.4 (1.26, 10.72)	3.6 (2.26, 6.61)	*0.767*
Median follow-up, months (range)		18.5 (4.4, 30.0)	13.0 (1.8, 18.1)	*0.032*
PTCY dose				
40 mg/kg, n		7		
29 mg/kg, n		10		
20 mg/kg, n			3	
10 mg/kg, n			11	
Neutrophil engraftment, days (range)		18(13, 38)	14 (12,33)	*0.251*
PLT engraftment, days (range)		32 (12, 61)	31(14, 45)	*0.735*
Graft failure, n (%)		1 (6)	0 (0)	*0.356*
PES, n (%)		13 (77)	12 (86)	*0.517*
Acute GvHD, n (%)		11 (65)	13 (93)	*0.062*
Grade 3 to 4 Acute GvHD, n (%)		5 (29)	8 (57)	*0.119*
HC, n (%)		2 (12)	5 (36)	*0.134*
Chronic GvHD, n (%)		5 (29)	8 (57)	*0.119*
CMV activity, n (%)		3 (18)	2 (14)	*0.597*
pneumonia, n (%)		6 (35)	4 (29)	*0.497*
Fungal infection, n (%)		3 (18)	4 (29)	*0.383*
Sepsis or bacteremia, n (%)		8 (47)	2 (14)	*0.058*
TRM, n (%)		2 (12)	1 (7)	*0.665*
Relapse, n (%)		6 (35)	0 (0)	*0.013*
Event-free survival, n (%)		9 (53)	13 (93)	*0.015*
RFS, mean (95%CI)		52.9 (29.18, 76.61)	92.9 (79.38, 100)	*0.047*
OS, mean (95%CI)		64.7 (41.96, 87.43)	92.9 (79.38, 100)	*0.281*

*PTCY* posttransplant cyclophosphamide, *SD* Standard deviation, *AML* Acute myeloid leukemia, *ALL* acute lymphoblastic leukemia; others, mixed lineage leukemia, *CR1* first complete remission, *CR2* second complete remission, *HLA* human lymphocyte antigen, *TNC* total nucleated cell, *CD34*^+^ CD 34^+^ cell counts, *PLT* platelet, *GvHD* graft-versus-host-disease, *HC* hemorrhagic cystitis, *CMV* Cytomegalovirus, *PES* peri-engraftment syndrome, *TRM* transplantation related mortality, *OS* overall survival, *RFS* relapse-free survival, *SE* Standard error, *95% CI* 95% confidence interval

### Immune cell recovery

Immunophenotypic analysis of T-lymphocyte (CD3^+^, CD3^+^CD4^+^, CD3^+^CD8^+^), B lymphocyte (CD19^+^) and NK cell (CD3^−^CD56^+^) reconstitution between days + 28 and + 35 was illustrated by percentages of immune cell counts in total white blood cell counts. At this period, CD3^+^CD4^+^-cell level was significantly lower in the high-dose PTCY group than in the low-dose group (20%, 95%CI (12%-27%) vs. 40%, 95%CI (31%-49%), *p* = 0.001). The median CD4^+^ to CD8^+^ ratio level was significantly lower in the high dose PTCY group than in the low dose PTCY group (0.62, range (0.04–6.46) vs. 2.04, range (0.29–5.05), *p* = 0.047). The total T-cell (*p* = 0.098), and CD3^+^CD8^+^-cell (*p* = 0.737) levels were not significantly different between the dosage groups. Additionally, the total T-cell (*p* = 0.925), CD3^+^CD4^+^-cell (*p* = 0.595), CD3^+^CD8^+^-cell (*p* = 0.539) levels, and the CD4^+^ to CD8^+^ ratio (*p* = 0.905) was not significantly different between the PTCY and non-PTCY groups. The differences of transfused NC (*p* = 0.812) and CD34^+^ (*p* = 0.361) were also not significant between the high-dose and low-dose PTCY groups.

## Discussion

In this study, we evaluated the safety and efficiency of PTCY as GvHD prophylaxis in UCBT for pediatric acute leukemia. The most important result of this study is that PTCY, at a dose of no more than 40 mg/kg, is a safe and effective strategy against PES and aGVHD in UCBT. This finding, discovered in UCBT data, differs from findings regarding haploidentical donor SCT and previous research about UCBT GvHD prophylaxis.

There are a few options for GvHD prophylaxis in UCBT. MMF has been suggested as a first-line choice [25, 26]. However, according to the results of this study, when MMF was used, the incidence of GvHD and PES were both high in our center though good survival has been presented. Therefore, an extra immunosuppression commencement was considered for the control of PES and PES proceeding aGvHD. PTCY is effective in haplo-HSCT and haplo-HSCT plus CB transplantation [18]. In this present study, PTCY is associated with lower incidences of PES, aGvHD, cGvHD, and HC in PTCY group, although the differences were not significant. In a haplo-HSCT study, both PTCY and ATG decreased GvHD and were shown effective in aGvHD prophylaxis [27]. However, the activation of viruses after ATG has been a concern in UCBT, which was why ATG was not the first choice when it comes to UCBT [17, 28, 29]. In another study, GvHD prophylaxis using MTX (10 mg/m^2^ for day + 1 and day + 3) after UCBT decreased the incidence of PES and severe aGvHD [1, 11]. However, the neutrophil engraftment rate was 84%, while the platelet engraftment rate was 81%. The engraftment rates in that study were so low that it might lead to TRM. Based on these findings, we adapted the PTCY treatment in combination with CsA/Tac and MMF as an innovative GvHD prophylaxis. OS and RFS were lower when PTCY was used. According to the multivariate analysis, the use of PTCY or not is not an independent risk factor for OS or RFS. In addition, PTCY did not significantly affect neutrophil and platelet engraftment time or graft failure incidence. Regarding infections, we observed increased sepsis or bacteremia incidence in the PTCY group, as well as the elevation of the rates of CMV activity and fungal infection. However, the infections did not contribute to an increased TRM. Therefore, PTCY, at a dosage of not more than 40 mg/kg, was shown to be safe in UCBT for pediatric acute leukemia.

Regarding the dosage of PTCY, according to previous studies, a single dose of PTCY (50 mg/kg on day + 3) was effective in preventing aGVHD [18], indicating that there may be room for further reduction in the dosage of PTCY [19]. In this study, we lowered the total PTCY dose to no more than 40 mg/kg. Since PTCY in this study belonged to low-dose category, CsA/ Tac and MMF were used as before. OS and RFS were lower in our high-dose PTCY group. Decrement in RFS was mostly attributed to the pre-transplantation remission status of CR2 according to the result of multivariate analysis. TRM, which was similar in the different doses of PTCY, was mostly attributed to graft failure. High-dose PTCY delayed neutrophil engraftment for up to 3 days. Patients with high-dose PTCY were more likely to get sepsis or bacteremia and pneumonia, but not CMV activity and fungal infection. Further evaluation of the efficiency of PTCY at different dosages was done by comparison in the immune and inflammatory reaction-related manifestations. High doses of PTCY decreased the incidences of PES, aGvHD, severe aGvHD, cGvHD, and HC. According to the results of the lymphocyte subset study, higher doses of PTCY (40 mg/kg or 29 mg/kg) presented a significantly lower CD4^+^ percentage, which was in accordance with previous report on the mechanism of PTCY inhibiting GvHD by limiting CD4^+^ subset proliferation in the early period of transplantation [30]. We assumed that PTCY of both 40 mg/kg and 29 mg/kg would be effective in inhibiting GvHD, but the effects on relapse merit future studies. Furthermore, the effects on severe infections of PTCY should be cautiously evaluated in upcoming studies. The impact of 20 mg/kg PTCY should be evaluated in the future as well.

There are several limitations to the present study. The inclusion periods for patients in the non-PTCY group and the PTCY group were different, which made the follow-up time for the PTCY group shorter. However, both groups had completed first-year evaluations, which may have mitigated the impact on the results reported. This was not a prospective randomized control study. However, the baseline characteristics were comparable between groups, except for CD 34^+^ cell counts of grafts. The most important bias may be the primary diseases. AML and ALL post-transplantation achieved different prognoses, which made it difficult to determine whether PTCY was beneficial in acute leukemia. CR2 status was shown to be the independent risk factor for relapse post-UCBT. CR2 patients are less likely to benefit from PTCY than CR1 patients. However, due to the limited number of CR2 patients included, it is difficult to determine whether CR2 patients (especially CR2 ALL) benefited from PTCY in UCBT. It would be worthwhile to conduct randomized controlled studies on different PTCY dosages in UCBT for different types of acute leukemia. Due to the small sample size and different doses in PTCY, we should further analyze and discuss the impact of the dose of PTCY in the future studies.

## Conclusion

The results of this study supported the safety and efficiency of PTCY as part of PES controlling and GvHD prophylaxis in single-unit UCBT for children with acute leukemia. The dose of 29 mg/kg or 40 mg/kg of PTCY presented more potential in GvHD prophylaxis in UCBT. Further study on the appropriate dose of PTCY in UCBT for acute leukemia is recommended.

## Supplementary Information

**Additional file 1.**

## Data Availability

The datasets used and/or analyzed during the current study are available from the corresponding author upon reasonable request.
