# Supplementary material for: Impact of posttransplant cyclophosphamide on the outcome of patients undergoing unrelated single-unit umbilical cord blood transplantation for pediatric acute leukemia
Source: BMC Cancer. 2022 Nov 18;22:1190. doi: 10.1186/s12885-022-10309-9 (PMC9675180; doi:10.1186/s12885-022-10309-9)
Supplement: Supplementary file 1 — Additional file 1. [file 12885_2022_10309_MOESM1_ESM.docx]

Supplementary Information 1. Spitizer Criteria for the diagnosis of Peri-engraftment syndrome.

The major criteria included: ①fever of >38.3℃ without identifiable infectious etiology, ② erythrodermatous rash involving 42% of the body surface area and not attributed to medication and ③ noncardiogenic pulmonary edema manifested as diffuse pulmonary infiltrates and hypoxia. The minor criteria included: ① hepatic dysfunction with either total bilirubin ≥2 mg/dL or transaminase levels greater than or equal to twice of the normal level, ② renal insufficiency (serum creatinine of greater than or equal to twice of the baseline value), ③ weight gain >2.5% of the baseline body weight and ④transient encephalopathy that was unexplained by other causes. A diagnosis of PES was established based on the presence of all three major criteria, or of two major criteria and one or more of the minor criteria, without clinical and pathological symptoms and signs of GVHD or infection.
